# Nerve-Sparing Modified Radical Hysterectomy for Severe Endometriosis and Complex Pelvic Pathology

**DOI:** 10.7759/cureus.9882

**Published:** 2020-08-19

**Authors:** Camran Nezhat, Kimsa Nguyen, Eliza Ackroyd, Robert A Roman, Anupama Rambhatla, Azadeh Nezhat, Atena Asiaii

**Affiliations:** 1 Center for Special Minimally Invasive and Robotic Surgery, Camran Nezhat Institute, Palo Alto, USA

**Keywords:** endometriosis, fibroids, complex pelvic pathology, modified radical hysterectomy, laparoscopy, minimally invasive surgery, robotic surgery

## Abstract

Background

Laparoscopic nerve-sparing modified radical hysterectomy with or without robotic assistance is known for its benefits as a definitive treatment for severe endometriosis. Undiagnosed endometriosis is common in patients with symptomatic fibroids or chronic pelvic pain. There are minimal studies that outline the safety and feasibility of nerve-sparing modified radical hysterectomy for other complex pelvic pathology in addition to endometriosis.

Objectives

The aim of this study is to evaluate the incidence of hospital readmission, intraoperative and postoperative complications, and long-term pain relief after laparoscopic nerve-sparing modified radical hysterectomy for severe endometriosis and complex benign pelvic pathology.

Study design

We performed a retrospective observational study of patients who underwent laparoscopic nerve-sparing modified radical hysterectomy with and without robotic-assistance with a high-volume minimally invasive endoscopic surgeon between November 2017 and December 2019.

Results

A total of 112 patients met the inclusion criteria. There were no cases of vaginal cuff dehiscence, venous thromboembolism, genitourinary system injury, gastrointestinal tract injury, vessel injury, nerve injury, sepsis, or death. Three patients required postoperative hospital admission for the management of umbilical cellulitis, acute blood loss anemia, and possible Addison’s crisis. Other postoperative complications included allergic reaction to adhesives (1.8%) and urinary retention (0.9%). All patients reported significant pain relief at the time of their postoperative visits. Three patients reported return of pain symptoms within the first seven months after surgery, with one requiring an additional surgery for persistent pain.

Conclusions

Laparoscopic nerve-sparing modified radical hysterectomy with or without robotic assistance is a safe and feasible alternative that provides long-term symptom relief in patients undergoing hysterectomy for a variety of indications.

## Introduction

Endometriosis is a whole body disease that affects gynecologic organs such as the uterus, fallopian tubes, ovaries, and extragenital structures, and can be managed expectantly, medically, or surgically [1-3]. It is a progressive disease, and despite thorough treatment, around 25% of patients undergoing surgical treatment of endometriosis will require subsequent surgery due to recurrence [4,5]. Definitive surgery for endometriosis consists of total hysterectomy, and bilateral salpingectomy with or without bilateral oophorectomy in patients who have completed childbearing.

Women with severe endometriosis or complex pelvic pathology refractory to medical or conservative surgical management will often seek definitive treatment with hysterectomy. The American College of Obstetricians and Gynecologists (ACOG) recommends laparoscopic hysterectomy as a preferred alternative for benign disease when vaginal hysterectomy is not indicated or feasible [6]. In cases of endometriosis and other complex pelvic pathology such as large uterine fibroids, ovarian cysts, and/or severe adhesive disease, laparoscopy is often necessary for thorough evaluation and treatment of the etiology of pelvic pain. Laparoscopy allows for improved anatomical visualization and more thorough treatment of various pathologies in the abdomen and pelvis, including treatment of extragenital endometriosis [7].

While extrafascial laparoscopic hysterectomy (class I) is commonly used for the definitive treatment of endometriosis, evidence suggests that modified radical hysterectomy in the setting of deep infiltrative endometriosis results in decreased pain recurrence [8]. Given this, we recommend modified radical hysterectomy to almost all patients with stage IV endometriosis who choose to undergo hysterectomy. Modified radical hysterectomy (class II) involves removal of the cervix, parametrium, and proximal 1-2 cm of the vagina at the time of hysterectomy [9]. This is less extensive than a classical radical hysterectomy (class III), which includes transecting the uterine arteries at their origin from the anterior division of the internal iliac, removing the upper half of the vagina, and routine pelvic lymphadenopathy. While classical radical hysterectomy is necessary for the treatment of most early stage cervical cancers to ensure complete excision of diseased tissue with clear margins, the close to 30% morbidity does not make it a good choice for the management of benign gynecologic diseases [10].

In a retrospective study in 2014 that included 52 patients with severe endometriosis who underwent laparoscopic nerve-sparing modified radical hysterectomy, we showed that it was a feasible, safe, and effective method for achieving long-term improvement in pain symptoms [11]. However, it is important to note that endometriosis can often be found co-existing with other complex pelvic pathologies. One study found that 87% of patients who underwent myomectomy or hysterectomy for symptomatic leiomyomas (defined as abnormal uterine bleeding) were found to have endometriosis on histology despite not having a prior diagnosis of endometriosis [12]. In addition, between 71% and 87% of women with chronic pelvic pain have endometriosis [13]. Given the high prevalence of undiagnosed endometriosis in patients with symptomatic fibroids or chronic pelvic pain and the known benefit of modified radical hysterectomy in the setting of severe endometriosis, we perform laparoscopic nerve-sparing modified radical hysterectomy with or without robotic assistance for the majority of patients with severe endometriosis and complex pelvic pathologies undergoing definitive treatment in our practice. The goal of this study is to examine the rates of intraoperative complications, postoperative complications, unanticipated postoperative hospital admissions, recurrence of pain, and need for reoperation to assess the safety and feasibility of performing laparoscopic nerve-sparing modified radical hysterectomy for severe endometriosis and other complex pelvic pathologies in an outpatient surgical setting.

## Materials and methods

We performed a retrospective chart review of all patients who underwent laparoscopic nerve-sparing modified radical hysterectomy with and without robotic-assistance for severe endometriosis and other complex pelvic pathology between November 2017 and December 2019. All patients had failed medical management and underwent hysterectomy by a single minimally invasive endoscopic surgeon. Patients were identified using coding and billing data for hysterectomy from our electronic medical records system. All patient information was de-identified and stored in an encrypted database for analysis. Our study was determined to be IRB exempt by Pearl IRB (Protocol #20-CNEZ-101).

All surgeries were performed in the following standard fashion. Patients received perioperative antibiotic prophylaxis as per ACOG guidelines and had placement of nasogastric/orogastric tube, sequential compression devices, Foley catheter, and uterine manipulator [14]. Abdominal entry was performed starting with abdominal insufflation with a Veress needle. A mapping needle was used to map the abdomen for possible intra-abdominal adhesions. Once pneumoperitoneum was established, a 10-mm umbilical bladed trocar was placed through the umbilicus. Three 5-mm accessory ports were subsequently placed in the left lower quadrant, right lower quadrant, and supra-pubic area under direct visualization [15]. Survey of the upper abdomen and pelvis was performed to identify extragenital endometriosis and identify any other abdominopelvic pathologies. Prior to initiating the hysterectomy, adhesiolysis and treatment of extragenital endometriosis were performed to restore normal anatomy using a combination of CO_2_ laser, bipolar electrosurgical device, ultrasonic energy device, and sharp and blunt dissection as appropriate.

Nerve-sparing modified radical hysterectomy was performed as described in our previous study [11]. Briefly, the round ligaments were desiccated and transected close to the deep inguinal ring of the pelvic sidewall. The vesicouterine peritoneum was incised and the bladder was mobilized using both blunt and sharp dissection. At this point, the “new space” can be visualized during laparoscopy. The “new space” is bordered medially by the vesicocervical-vaginal ligament (also known as the bladder pillar), distally by the bladder, anteriorly by the remaining intact portion of the anterior leaf of the broad ligament, laterally by the obliterated umbilical artery, and posteriorly by the parametrium where the ureter traverses to the bladder [16,17]. The utero-ovarian ligament was desiccated and transected if oophorectomy was not planned. Bilateral ureterolysis was performed with dissection from the pelvic brim to the bladder and exploration of tunnels of Wertheim bilaterally. Intraoperative ureteral stents were not routinely utilized. We utilized hydrodissection to lateralize the ureters to protect them from injury during the operation. The superior vesical arteries were preserved when possible. The rectovaginal space was then further developed and the superior portion of the bilateral uterosacral ligaments was excised. The uterine arteries were skeletonized, desiccated, and transected beside and medial to the ureter. The colpotomy was then performed, with any obvious endometriosis involving the paracolpos and rectovaginal septum removed en bloc. After removal of the specimen vaginally, the vagina was closed laparoscopically using barbed suture in a running fashion.

Most patients were discharged home on the day of surgery once able to tolerate oral intake, ambulate, and void spontaneously. Patients received prophylactic cephalexin 500 mg twice daily for five days postoperatively per our practice protocol. They were seen for a postoperative visit four to six weeks after surgery. Patients were instructed to limit heavy lifting and sexual activity for at least eight weeks after hysterectomy.

All surgical and postoperative complications were recorded including known common complications of hysterectomy, including infection, venous thromboembolism, genitourinary and gastrointestinal tract injury, vessel injury, urinary retention, excessive bleeding, nerve injury, and vaginal cuff dehiscence [18,19]. Postoperative hospital admission was determined by a review of office and hospital online medical records. Pain relief was determined by the patient's verbal report at postoperative and follow-up appointments that were reviewed up to two years following surgery.

## Results

A total of 112 patients were included in this study, of whom 90 underwent laparoscopic nerve-sparing modified radical hysterectomy and 22 underwent laparoscopic nerve-sparing modified radical hysterectomy with robotic assistance. Average uterine specimen weight was 99.7 grams. Patients included in our study had an average age of 40.9 years (range: 25-62 years) and an average BMI of 26.1 with a standard deviation ±5.6. The most common presenting symptoms were pelvic pain (73.2%), dysmenorrhea (52.7%), menorrhagia (31%), and dyspareunia (33%). After obtaining a full history, all patients were assessed with a physical examination and ultrasound in the office to establish a diagnosis. Preoperative diagnoses are listed in Table 1. Of the patients, 49% had at least one prior surgery for the treatment of endometriosis. Additional procedures including cystoscopy, salpingectomy, oophorectomy, cystectomy, proctoscopy, lysis of adhesions, and hernia repairs were performed if indicated (Table 2). Of note, 68 (60.7%) patients had a history of prior laparoscopy, 26 (23.2%) had a history of cesarean delivery, 17 (15.2%) had a history of laparotomy, and 7 (6.25) had no prior history of abdominal surgery.

**Table 1:** Patient Characteristics

Preoperative Diagnosis	n (%)
Known or Presumed Endometriosis	100 (89.3)
Chronic Pelvic Pain	82 (73.2)
Chronic Abdominal Pain	76 (67.9)
Abnormal Uterine Bleeding	52 (46.4)
Adenomyosis	29 (25.9)
Fibroids	25 (22.3)
Adnexal Mass	6 (5.4)
Pelvic Mass	6 (5.4)
Prior Surgeries	n (%)
Endometriosis	55 (49.1)
Cesarean Delivery	26 (23.2)
Cystoscopy	17 (15.2)
Laparotomy	17 (15.2)
Appendectomy	16 (14.3)
Salpingectomy	14 (12.5)
Proctoscopy	10 (8.9)
Oophorectomy	8 (7.1)

**Table 2:** Surgical Procedures and Postoperative Complications

	n (%)
Adjunctive Procedures	
Cystoscopy	111 (99.1)
Proctoscopy	106 (94.6)
Bilateral Salpingectomy	88 (78.6)
Lysis of Adhesions	32 (28.6)
Unilateral Oophorectomy	26 (23.2)
Bilateral Oophorectomy	16 (14.3)
Unilateral Salpingectomy	13 (11.6)
Hernia Repair	5 (4.5)
Postoperative Complications Not Requiring Hospital Admission	
Allergic Reaction to Adhesives	2 (1.8)
Urinary Retention	1 (0.9)
Hospital Admission < 30 Days Postoperative	
Vaginal Cuff Bleed with Acute Blood Loss Anemia	1 (0.9)
Umbilical Cellulitis	1 (0.9)
Hospital Admission < 90 Days Postoperative	
Suspected Addison's Crisis	1 (0.9)

There were no intraoperative complications. Maximum estimated blood loss was 350 cc, and no patients required intraoperative blood transfusions. Of the 112 patients, 10 (8.9%) were admitted for observation immediately following surgery and all were discharged within 24 hours in stable condition. Three (2.7%) patients had minor postoperative complications that were managed in the outpatient setting (Table 2). Of the 112 patients, seven (6.3%) presented for urgent postoperative evaluation in the office or emergency department (ED) setting with one of the following: severe nausea, severe abdominal pain, bladder pain, allergic reaction to surgical skin glue, umbilical cellulitis, heavy vaginal bleeding, and possible Addison’s crisis.

Of those who presented for evaluation, three (2.7%) patients were admitted to the hospital. One patient with umbilical cellulitis was admitted eight days postoperatively for IV antibiotics and discharged on hospital day two. Another patient with heavy vaginal bleeding presented 10 days postoperatively to the ED and was diagnosed with acute blood loss anemia, requiring two units of packed red blood cell transfusion and a diagnostic laparoscopy to evaluate for the source of bleeding. Laparoscopic findings included slow bleeding from surgical sites and the lateral edge of the vaginal cuff, which was noted to be intact. She was admitted for observation and discharged the next day in good condition. Another patient, with a known history of Addison’s disease, presented to the ED 31 days postoperatively and was admitted to the hospital for three days for an endocrinology evaluation in the setting of suspected Addison’s crisis. There were no cases of vaginal cuff dehiscence, venous thromboembolism, genitourinary system injury, gastrointestinal tract injury, vessel injury, nerve injury, sepsis, or death in this population.

All patients reported significant pain relief per verbal report at the postoperative visit. Of the 70 patients who had a follow-up visit within six months of surgery, two (2.5%) patients reported recurrence of symptoms at two and three months, respectively. Of the 24 patients who had a follow-up between six months and one year of surgery, one (5.6%) patient reported recurrence of symptoms at seven months. Of the 18 patients who had a follow-up between one year and two years after surgery, no patients reported pain recurrence. All three patients who experienced a recurrence of pain had endometriosis and one subsequently underwent bilateral oophorectomy, with no recurrence of symptoms 16 months after her reoperation.

## Discussion

Nezhat et al. reported the first laparoscopic radical hysterectomy with aortic and pelvic node dissection in 1989, and in 2014 they reported a case series of 52 patients undergoing laparoscopic modified radical hysterectomy for complete excision of severe endometriosis [11,20]. Complete excision of endometriotic lesions including deep infiltrative lesions is a best practice for the treatment of endometriosis and decreasing the need for future reoperation [21]. Performing a modified radical hysterectomy in cases of severe endometriosis in place of an extrafascial hysterectomy can better ensure that all affected tissue are excised at the time of hysterectomy, leading to decrease in pain recurrence in patients with endometriosis.

There are few studies that review the use of radical hysterectomy as a treatment for severe endometriosis. One study compared laparoscopic versus laparotomic radical en bloc hysterectomy and colorectal resection in 29 patients who underwent surgery for extensive endometriosis between April 2001 and July 2008 [22]. This study evaluated the feasibility, quality of life, and urinary function in patients postoperatively. Quality of life was determined using the Short-Form 36 Questionnaire, and urinary function was evaluated using the International Prostate Score Symptoms and Bristol Female Lower Urinary Tract Symptoms questionnaires. Patients in both groups reported similar improvements in symptoms and quality of life following surgery, and the authors concluded that radical en hysterectomy and colorectal resection should be offered to appropriate patients.

More recently, Melnyk et al. examined 333 patients to determine if there was a difference in intraoperative outcomes in endometriosis patients with and without obliterated cul-de-sacs undergoing laparoscopic hysterectomies between 2012 and 2016 [23]. They found that patients with stage IV endometriosis who also had an obliterated cul-de-sac required laparoscopic modified radical hysterectomy in addition to other intraoperative procedures compared to those who had stage IV endometriosis without an obliterated cul-de-sac. They found 1% of patients with stage IV endometriosis had complications including pulmonary embolism and 3% had postoperative skin infections, though only 66% of their patients with stage IV endometriosis underwent modified radical hysterectomy and the remaining 34% underwent extrafascial hysterectomy. Of their patients with stage I through III endometriosis, 74% underwent an extrafascial hysterectomy, 25% underwent a modified radical hysterectomy, and 0.4% underwent a supracervical hysterectomy. These patients experienced complications including skin infection (3%), bladder injury (<1%), bowel injury (<1%), blood transfusion (<1%), conversion to open (<1%), and cuff cellulitis (<1%). Pain recurrence and need for reoperation were not examined in this study. Despite our higher rate of modified radical hysterectomies, there were very few postoperative complications that were each in <1% of the patients, with the exception of allergic reaction to adhesives occurring in 1.8% of patients. Overall, we agree with their recommendation that patients with severe endometriosis should be referred to specialists familiar with this surgery due to the difficulty of these cases.

The importance of a nerve-sparing approach during surgical treatment of endometriosis was reviewed in a study by Ceccaroni et al. in 2012 [24]. They reviewed outcomes from 126 patients, of whom 61 were treated with nerve-sparing radical excision of pelvic endometriosis and 65 were treated with the classical laparoscopic endometriosis excision. This study found that patients in the nerve-sparing group experienced significantly lower rates of severe neurologic pelvic dysfunction with comparable rates of endometriosis recurrence and intraoperative, perioperative, or postoperative complications. With nerve-sparing techniques, surgeons hope to minimize rectal, urinary, and sexual function side effects [25,26].

Whether or not to perform oophorectomy at the time of hysterectomy for patients with endometriosis is controversial. Studies have shown that when ovaries are preserved at the time of hysterectomy, there is a sixfold increased risk of recurrent pain and an eightfold increased risk of reoperation [21,27]. While bilateral oophorectomy may decrease the recurrence risk of endometriosis, it can lead to serious consequences including increased all-cause mortality, risk of cardiovascular disease, and osteoporosis. Interestingly, our rates of pain recurrence and reoperation are lower than those previously reported despite ovarian preservation in more than half of our patients [27,28]. We suspect that this may be attributed to performing modified radical hysterectomy instead of extrafascial hysterectomy. Our study had only one patient requiring repeat surgery for endometriosis treatment, though we cannot account for patients who may have presented to outside facilities for care. While our study included more diverse indications for hysterectomy, 89% had a preoperative diagnosis of either known or presumed endometriosis.

The overwhelming majority of patients in this study reported good pain relief with low unanticipated postoperative hospital admittance and low reoperation rates; however, this study is not without its limitations. The number of patients in our sample is small (n = 112) with no control group, which may contribute to bias in our results. Given the retrospective observational study design, the patient sample is especially vulnerable to selection bias. For example, the average uterine specimen weight was <100 grams in this particular population; if patients with larger uteri were included, postoperative complication rates may have been different. Furthermore, since many patients in our practice travel from far distances to seek care, some may have presented with complications, pain recurrence, and need for reoperation at local facilities, resulting in an underestimation of these findings in our study. Another limitation is the binary assessment of pain by verbal report in our study; the use of verified pain scales would better standardize pain assessments. Additionally, the short-term follow-up of two years may prevent findings related to long-term pain recurrence following surgery. Lastly, the surgeries were performed by a high-volume surgeon very familiar with performing nerve-sparing modified radical hysterectomy. These limitations may hinder the generalizability of our findings to the greater population. Future studies can include a randomized controlled trial of patients undergoing extrafascial hysterectomy compared to those undergoing modified radical hysterectomy to compare complication rates, short- and long- term symptom relief, and need for reoperation.

## Conclusions

This study demonstrates that laparoscopic nerve-sparing modified radical hysterectomy with or without robotic assistance is a safe and feasible alternative that provides long-term symptom relief in patients undergoing hysterectomy for a variety of indications. Given the high prevalence of undiagnosed endometriosis and improved patient outcomes with modified radical hysterectomy in those with endometriosis, it follows that patients who undergo hysterectomy for diagnoses other than endometriosis may also have improved outcomes after undergoing modified radical hysterectomy as compared to extrafascial hysterectomy. The improved outcomes may also mitigate the need for future oophorectomy, which is more common after hysterectomy for endometriosis. We recommend that patients should be referred to an experienced gynecologic surgeon for consideration of nerve-sparing modified radical hysterectomy in cases of severe endometriosis and complex pelvic pathology.
